# The Effect of Magnetic Field Gradient and Gadolinium-Based MRI Contrast Agent Dotarem on Mouse Macrophages

**DOI:** 10.3390/cells11050757

**Published:** 2022-02-22

**Authors:** Priyanka Chanana, Ahmed Uosef, Nicole Vaughn, Martha Suarez-Villagran, Rafik M. Ghobrial, Malgorzata Kloc, Jarek Wosik

**Affiliations:** 1 The Houston Methodist Research Institute, Houston, TX 77030, USA; inkachanana@gmail.com (P.C.); auosef@houstonmethodist.org (A.U.); NicoleReneVaughn@gmail.com (N.V.); rmghobrial@houstonmethodist.org (R.M.G.); 2 Department of Surgery, The Houston Methodist Hospital, Houston, TX 77030, USA; 3 Physics Department, University of Houston, Houston, TX 77204, USA; mayosuvi@gmail.com; 4 Texas Center for Superconductivity, University of Houston, Houston, TX 77204, USA; 5 MD Anderson Cancer Center, Department of Genetics, The University of Texas, Houston, TX 77030, USA; 6 Electrical and Computer Engineering Department, University of Houston, Houston, TX 77204, USA

**Keywords:** magnetic field gradient, MRI, gadolinium, macrophage, polarization, mitochondria, Golgi complex, ER

## Abstract

Magnetic resonance imaging (MRI) is widely used in diagnostic medicine. MRI uses the static magnetic field to polarize nuclei spins, fast-switching magnetic field gradients to generate temporal and spatial resolution, and radiofrequency (RF) electromagnetic waves to control the spin orientation. All these forms of magnetic static and electromagnetic RF fields interact with human tissue and cells. However, reports on the MRI technique’s effects on the cells and human body are often inconsistent or contradictory. In both research and clinical MRI, recent progress in improving sensitivity and resolution is associated with the increased magnetic field strength of MRI magnets. Additionally, to improve the contrast of the images, the MRI technique often employs contrast agents, such as gadolinium-based Dotarem, with effects on cells and organs that are still disputable and not fully understood. Application of higher magnetic fields requires revisiting previously observed or potentially possible bio-effects. This article focuses on the influence of a static magnetic field gradient with and without a gadolinium-based MRI contrast agent (Dotarem) and the cellular and molecular effects of Dotarem on macrophages.

## 1. Introduction 

Magnetic resonance imaging (MRI) is widely used in diagnostic medicine to visualize the anatomy, structure, and physiology (for example, blood flow) of patients’ organs. MRI is based on a nuclear magnetic resonance phenomenon in which, when certain nuclei (for example, ^1^H or ^13^C) are placed in a static magnetic field, resonant absorption and emission of electromagnetic radiofrequency (RF) energy occur [1]. Because MRI does not use ionizing radiation, it is believed to be much safer for the patient than radiation/X-ray-based scanning techniques [2]. However, besides the static magnetic field employed to polarize nuclei spins, MRI needs fast-switching magnetic field gradients for generation of temporal and spatial resolution and also RF electromagnetic signals to manipulate the spins’ orientations. Both magnetic static and electromagnetic RF fields interact with human tissue and cells. Early studies of bio-interactions of different kinds of magnetic fields with living organisms reported a lack of significant harmful effects of a static magnetic field in contrast to some safety issues such as thermal effects induced by RF electromagnetic transmit signals [3,4,5].

However, on the molecular level, the data related to the magnetic field interaction with the cell organelles are often inconsistent and contradictory [6,7]. One of the reasons is the very small diamagnetism of cells/tissue that, even in the case of a relatively strong magnetic field, the maximum magnetic field generated force acting on cells is comparable with the gravitational forces. Human body functions are evolutionarily adjusted to the presence of the Earth’s gravity and also to forces resulting from human movement. 

MRI as a technique suffers from an inherently small signal-to-noise ratio (SNR). The SNR as well as the spectral dispersion and susceptibility contrast can be improved by increasing the static magnetic field strength of magnets. Such a trend can clearly be seen in clinical imaging, where the field strength was increased from 1.5 T in early scanners to 7 T in recently introduced state-of-the art scanners [4,8]. Higher fields, such as 9.4 T and 11.7 T, have already been tested on humans, and even for rodents, ultra-high fields above 20 T have started to be employed [9,10].

Furthermore, to improve the visibility and enhance the details and clarity of an organ’s structure, the MRI technique often employs contrast agents given to the patient orally, injected into the joints, or intravenously. Due to the fact of their magnetic properties, contrast agents are changing the relaxation times of surrounding nuclei, resulting in local changes in the image contrast. The most widely used intravenous contrast agents are based on paramagnetic gadolinium [11]. 

Gadolinium is a ductile, silvery-white, rare earth metal that can form very toxic Gd^3+^ ions in solution. It exhibits a strong paramagnetism due to the seven unpaired electrons in the inner 4f shell, and this paramagnetism is maintained even if it is bonded to another molecule or anions. Injected Gd^3+^-based contrast agents shorten the locally longitudinal relaxation time T_1_ that results in a much brighter, better contrasted T_1_-weighted MRI image. To prevent Gd^3+^ toxicity, the gadolinium used as an MRI contrast agent is chelated, i.e., is bound to a sequestering carrier ligand. Depending on the type of ligand, gadolinium-based contrast agents are divided into two categories: (a) the linear, where the flexible open chain of an organic molecule wraps around a central paramagnetic Gd^3+^ ion, or (b) the macrocyclic, when the ligand is preorganized into a rigid molecular cage that chelates the ion [12,13]. Another parameter, which affects the stability of gadolinium-based contrast agents is whether the linear coordinating molecule or the chelating ligand is ionic or non-ionic [14,15,16]. The ionic ligands provide very strong binding between the Gd^3+^ and negatively charged carboxyl groups, while the non-ionic ligands have weaker binding between the Gd^3+^ and amide or alcohol functional groups [13,17]. The most stable and, thus, potentially least toxic are the ionic-macrocyclic gadolinium-based contrast agents such as gadoterate meglumine (Gd-DOTA, also called Magnescope^®^ in Japan and Dotarem^®^ in the United States and other countries), and the least stable are the nonionic linear chelates such as gadodiamide and gadoversetamide [11,18]. The gadolinium-based contrast agent Dotarem used in this study belongs to the ionic-macrocyclic category. With an exponential increase in the diagnostic use of MRI in hospitals and clinics, and with some patients receiving many MRI procedures during their lifetime, it has become clear that the stability of a gadolinium-based contrast agent is an important factor that is linked to serious side effects and pathogenesis of contrast-agent-based MRI. It has been shown that the free gadolinium ions released from the nonionic linear chelates aggregate in various organs and tissues and cause fibrosis and other side effects [19,20]. As a remedy, ionic-macrocyclic chelates have been recommended for clinical use. 

However, many studies show that even the ionic-macrocyclic chelates are not completely safe and that the gadolinium can cross the brain–blood barrier and accumulate in the brain [21,22,23,24]. These troublesome findings led the US Food and Drug Administration to recommend limiting gadolinium-based contrast agents to clinical necessities only when the benefits outweigh the risks and urge practitioners to reassess the repeated use of gadolinium contrast agents.

There is also an increasing concern about the potential adverse health effects of a very strong magnetic field and static magnetic field gradients produced by MRI magnets. Superconducting magnets used in MRI generate the strongest static magnetic fields to which humans have ever been exposed. Numerous early investigations focused on the bioeffects of magnetic fields and their interactions with living organisms. They were not fully conclusive but mostly showed a lack of adverse effects, even when a relatively wide range of magnetic field strength was applied [5,25]. The main reason behind these either negative or non-repeatable findings is living organisms’ very small diamagnetic susceptibility. In addition, in some static magnetic field studies, even if the influence and effects were observed, they were below the point of significance, especially in cases where the magnetic field generated forces were comparable or smaller than the forces introduced by the Earth’s gravity. In most cases, living organisms develop mechanisms stabilizing cells and cells’ functions in the presence of gravity or movement-related mechanical forces. 

However, the current trend in the MRI industry to enhance the resolution and sensitivity of the technique by increasing the magnetic field strength, which requires the design and fabrication of ever-stronger superconducting magnets, significantly increases chances that the mechanical forces generated by the presence of a static magnetic field will be noticeable and potentially harmful. In addition, in recent years, the use of paramagnetic or ferromagnetic contrast agents has become more common to further enhance the contrast and specificity of MRI. Such additions, due to the fact of their magnetic properties, are expected to increase magnetic field-related mechanical forces generated by uniform or/and nonuniform magnetic fields. This strongly implies that the studies of static magnetic field interaction with living organisms should be revisited.

To address the issue of static magnetic field interaction and its effect on living cells, we studied the effects of applying a nonuniform magnetic field with and without a paramagnetic contrast agent (i.e., Dotarem) on cultured mouse macrophages. We also studied the cellular and molecular effects of Dotarem on the macrophages.

We started our studies on macrophages many years ago when we discovered that they are crucial for the development of long-time (chronic) rejection of transplanted organs. We discovered that the interference with the actin cytoskeleton and RhoA pathway-elongated macrophages changed the distribution and functions of their organelles and inhibited chronic rejection. Because the RhoA pathway is also regulated by mechanotransduction, we initiated studies on the effect of the magnetic field gradient on macrophages.

## 2. Material and Methods

### 2.1. Magnetic Field Generation and Permanent Magnets’ Configuration

**Figure 1:**
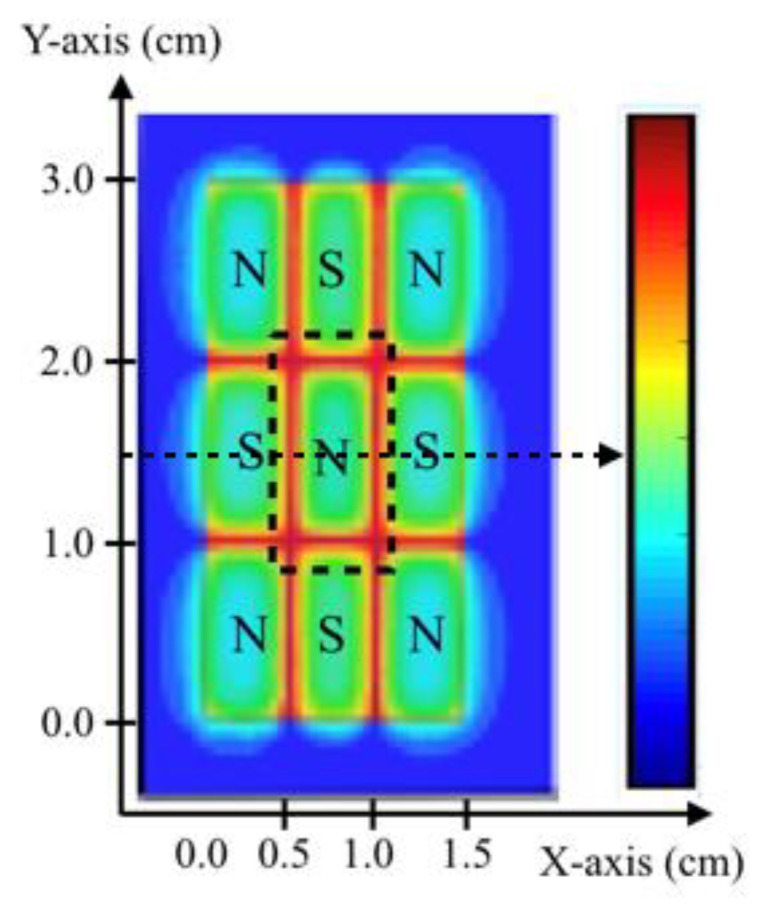
Configuration of rare earth permanent magnets which allows for a symmetric nonuniform field around the central magnet. A sketch of the array of the 9 magnets with an antiparallel N–S pole configuration is shown. The calculated magnetic force, ΔχB_z_$\frac{\partial B}{\partial z}$, component on the *x-* and *y*-planes confirms the uniform magnitude of the force along all adjacent interfaces of the central magnet (red color). The dashed lines show the area of interest in our experiments.

For magnetic field experiments with cultured macrophages, an array of nine rare earth permanent (NdFeB) rectangular magnets (ND105236; Digi-Key, Thief River Falls, MN, USA) was designed and assembled. All magnets were axially magnetized and had the same dimensions: 5 mm × 10 mm × 1.9 mm. Magnetization direction was perpendicular to the 5 × 10 mm surface of each magnet, and all adjacent pairs had an antiparallel NS–SN alignment. The magnets’ configuration, as depicted in Figure 1, allowed for the creation of a symmetric magnetic field/magnetic gradient pattern above the central magnet.

Such an arrangement, due to the fact of its antiparallel NS–SN configuration, creates a steep-wall well of the magnetic force lines around the central magnet. That is why only macrophages in the area above the central magnet, as marked with the dashed lines in Figure 1, were investigated in our study. The 5 × 10 mm long magnet sides were polished to minimize gaps between adjacent magnets. The whole structure of nine magnets was tightly framed using a G-10 fiber epoxy custom holder.

**Figure 2:**
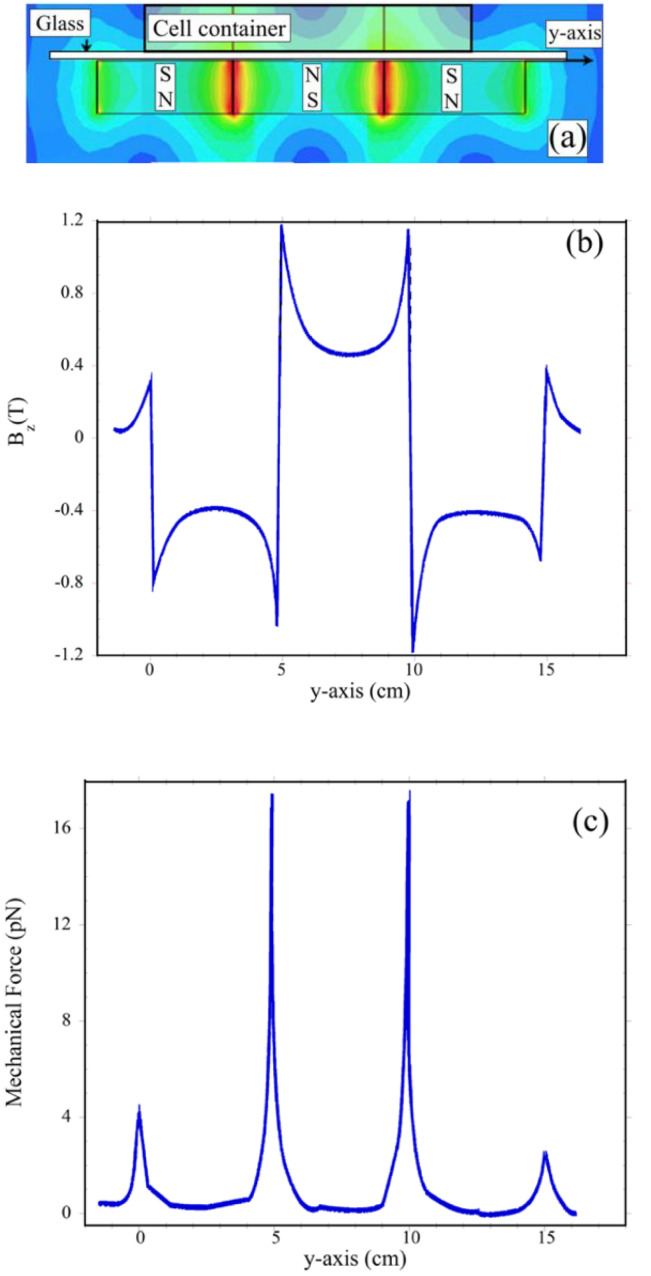
Magnetic field induction B and magnetic field-induced mechanical force patterns. (**a**) Sketch of the experimental configuration of the magnets and the cells’ container position. The dimensions of the glass plate, magnets, and container are not to scale. The theoretically calculated magnetic-field induction vector B_z_ in the *yz*-plane is 2D plotted along the *y*-axis; (**b**) shows magnetic-field induction magnitude of the B_z_ component along *y*-axis; (**c**) The mechanical force acting on a cell along the *y*-axis is shown. Calculations were conducted for three very closely aligned 5 mm wide, 10 mm long, and 1.9 mm thick magnets configured in opposite magnetic polarization (NS–SN) directions.

The magnetic field, magnetic field gradient, and magnetic force distribution were simulated using the Ansoft Maxwell (ANSYS, Canonsburg, PA, USA) and MATLAB software packages. The magnets used for experiments had the following specification: remanence magnetization B_r_ (min), which as a measure the magnetic field strength was equal to 1.24 T; coercivity H_cB_ (induction curve) of 950 kA/m; coercivity H_cJ_ (polarization curve) of 1750 kA/m. The material parameters, simulated magnets’ geometry, and field boundary conditions closely mimicked the experimental configuration (Figure 1) and used materials. Plots of the calculated magnetic field, B_z_, and mechanical force, F_z_, components along the *y*-axis are shown in Figure 2b,c.

### 2.2. The Magnetic Field Gradient Created a Mechanical Force Acting on Macrophages

The nature and magnitude of the interactions of the electric and magnetic fields with cells or tissue depend on the polarizations produced by such fields. The ability to induce polarization is characterized by electric and magnetic field susceptibilities. There were significant differences between the interactions of these fields with cells/tissue, because for a typical tissue, the electric field susceptibility is 10^5^–10^6^ larger than the magnetic field susceptibility and, as a result, the electric field can cause significant cells/tissue damage, whereas magnetic field interactions with cells/tissue are relatively weak due to the weak diamagnetism of the cells. Magnetic field-induced forces are, in most cases, smaller or only comparable with gravitational forces. In principle, when a diamagnetic object is suspended in a nonmagnetic medium, it is expelled by the magnetic field. However, the buffer or plasma medium can be either more or less diamagnetic than the cells. In such a case, the response of cells to the presence of a magnetic field depends on the difference between susceptibilities of the cell and medium. 

The magnitude of the magnetic force is dependent on the product of the magnetic induction ~$\;\vec{B}$ (in T units) and the magnetic field gradient ∇*B* (in T/m units). When a biological object is placed in a magnetic field, the energy U parameter, which depends on magnetic moment, m, and magnetic field induction, $\vec{B}$ ($U=-\left(1/2\right)\vec{m}\cdot \vec{B}$), can be used to describe forces acting on this object [26]. The formula describing the translational force as $\vec{F}=-\nabla U$ determines the sign of cells and medium susceptibilities difference, Δ*χ*, and it can be expressed as:

(1) $$\vec{F}\left(x,y,z\right)=\frac{\left(\chi_{medium}-\chi_{cell\;}\right)V_{cell}}{\mu_{0}}\vec{B}\cdot \left(\nabla \vec{B}\right)=\frac{\Delta \chi V_{cell}}{\mu_{0}}\vec{B}\cdot \left(\nabla \vec{B}\right)$$

where ∇ is the del operator, and *V_cell_* is the cell volume. Δ*χ* = (*χ*_cell_ − *χ*_medium_) describes the difference in magnetic susceptibilities between the cell and the surrounding buffer medium. 

**Figure 3:**
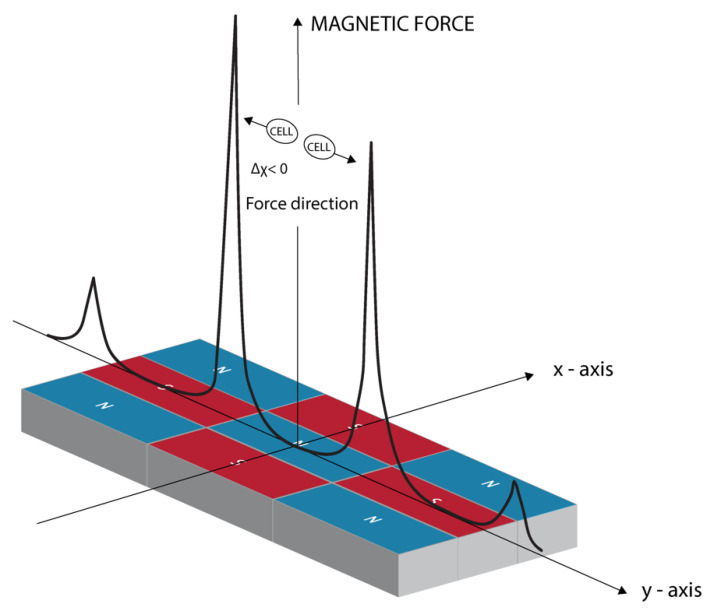
Magnetic field gradient created mechanical force direction. A calculated plot along the *y*-axis of the magnetic field-induced mechanical force, F_z_, component. For the case when the medium surrounding the cells is more diamagnetic than the cells (Dc < 0), cells are attracted to stronger and more nonuniform magnetic field areas as marked in this sketch.

In a nonuniform magnetic field, cells that are less diamagnetic than the surrounding plasma will be attracted to the higher field regions. The force vector will be reversed if the cells are more diamagnetic than the surrounding medium, because cells will be repelled from the higher field area. Figure 3 shows the force direction acting on macrophages along the *y*-axis [27] that was observed in our experiments. The situation is similar to the one reported for red blood cells in nonuniform fields [5]. The presence of iron atoms in hemoglobin makes the red blood cells slightly less diamagnetic than surrounding plasma; as a result, in a nonuniform magnetic field, the cells will move relative to the surrounding plasma toward regions of strong magnetic fields.

It is analogous to the ordinary effect of buoyancy observed when objects float or sink in a liquid medium under the influence of gravity. Gravity force, $\vec{F}$, in such a case is equal to $m\vec{g}$. Thus, for magnetic translational force, $\vec{B}\cdot \nabla \vec{B}$ plays the role of the standard acceleration factor, $\vec{g}$, due to the presence of gravity; the Δχ*V_cell_*/*μ*_0_ role is equivalent to the mass, m.

For biological materials, both the susceptibilities are usually negative in all directions and are of the order of 10^−5^ in SI units. Almost all human tissues are diamagnetic and have susceptibilities in a narrow range of approximately 20% from the susceptibility of water, which is approximately −9 × 10^−6^.

Application of the nonuniform magnetic field can introduce the translational mechanical force applied to the cell and, as a result, can create some internal forces that propagate into the cell via its cytoskeleton. For the actin filament network, the magnitude of such forces is believed to be in the range from 10 to several nN [28,29]. Another force is the adhesion force exerted by the cell on a substrate to which it is attached through focal adhesions. This force typically ranges from 1 to 100 nN [30,31].

For comparison purposes, a single protein’s unfolding and stretching process is known to require 2–10 pN [32]; while stretching of a complete actin filament, which consists of many protein monomers, requires a much larger force of approximately 10 nN [33,34]. The translational forces acting on cells can reach approximately 10 pN produced by a nonuniform magnetic field created by a set of 1.24 T rare earth permanent magnets [27,35].

### 2.3. Macrophage Culture on Magnets

**Figure 4:**
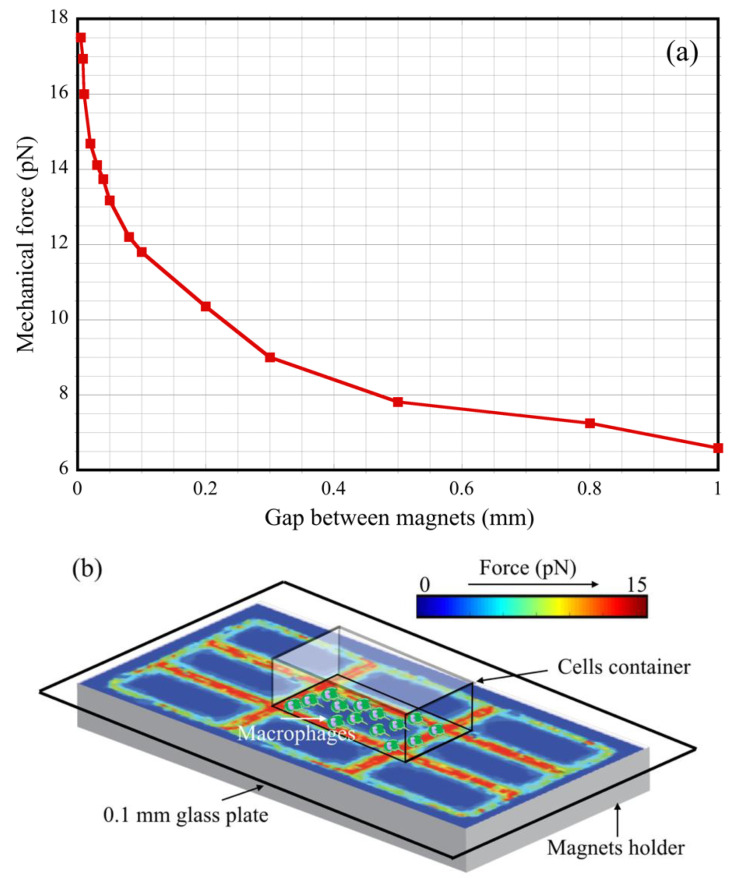
Mechanical force dependence on the magnet separation gap and the distance from magnet interface. (**a**) Calculated force acting on the macrophages shown as a function of the gap size between the magnets for a 0.1–1.0 mm range; (**b**) the cell container with macrophages is sketched above the area of interest.

Mouse peritoneal macrophages were isolated from C57BL/6 (H-2b) mice. Macrophages were grown in Dulbecco’s modified Eagle’s medium (DMEM) supplemented with 10% heat-inactivated fetal bovine serum, 100 U/mL penicillin, and 100 µg/mL streptomycin with the addition of 10 ng/mL of macrophage colony-stimulating factor (MCSF, PeproTech) as described previously [36] without or with 20 mM of the MRI contrast agent Dotarem (cat. NDC67684-2001-3, Guerbet, Raleigh, NC, USA) in the custom-made chamber slides in which a bottom 1 mm thick standard microscope slide was replaced with a thin (0.1 mm thick) cover glass. These modified chambers were placed on top of the magnets. Figure 4b shows a sketch of such an experimental configuration. The thin (i.e., 0.1 mm) cover glass at the bottom of the chamber allowed for a decrease in the distance between the cells and the magnet. Simulations of the translational force created by the magnetic field gradient as a function of the cell–magnet distance confirmed that there was only a small reduction in the force related to the increased distance. It is also important to keep the permanent magnets as close as possible to each other in these kinds of experiments. The surfaces of all magnets were planarized and polished with a few-micron sandpaper. The dependence of the translational force at the N–S interface on the magnets’ separation gap is plotted in Figure 4a.

The macrophages in the chamber slides (0.4 × 10^6^/slide) were fixed in 4% formaldehyde in PBS with 0.05% Triton X-100. After washing in PBS–Tween 20, fixed cells were blocked in casein blocking buffer (BioRad, USA) with 0.05% Tween 20 and incubated in blocking buffer with rhodamine–phalloidin (Thermo Fisher Scientific, Waltham, MA, USA, cat. R415) (2 μL of methanolic solution/500 mL) to visualize actin, overnight at 4 °C. Subsequently, after washing in PBS–Tween 20, cells were mounted in ProLong^®^ Diamond Antifade with DAPI (Fisher Scientific, USA, cat. P36962) and observed under a Nikon fluorescence microscope. Additionally, mouse peritoneal macrophages grown on the magnet setup described in two previous studies [27,35] were immunostained with mitochondrial marker anti-COX4 antibody (cat. 459600, Invitrogen, Carlsbad, CA, USA) according to the protocol described below for the RAW 264.7 macrophages.

### 2.4. Mice

Mice were housed and used according to the Methodist Hospital Research Institute’s animal care protocol and NIH standards were used in concordance with the “Guide for the Care and Use of Laboratory Animals” (DHHS publication No. (NIH) 85-23 Revised 1985), the PHS “Policy on Humane Care and Use of Laboratory Animals”, and the NIH “Principles for the Utilization and Care of Vertebrate Animals Used in Testing, Research and Training”. All studies were performed according to the animal protocol AUP-0317-0006 (IS00003962), entitled “Tolerance Induction in a Rodent System”, approved by the Houston Methodist Institutional Animal Care and Use Committee. 

### 2.5. Dotarem Treatment of RAW 264.4 Cells

Dotarem (cat. NDC67684-2001-3, Guerbet, Raleigh, NC, USA) was used at a 20–30 mM concentration. The 20 mM dose was chosen as the one used on cells in several in vitro studies (for example, Russell et al. [37]). The used dose was higher than that given to humans; however, in many human tissues and cells, Dotarem accumulates, especially after multiple MRI scans with the contrast agent, resulting in a high intracellular concentration. The experiments were performed on the Abelson murine leukemia transformed mouse macrophages RAW 264.7 (ATCC TIB-71) from the ATCC. Because it is known from the literature [38] that RAW 264.7 cells change properties around passage 15, we used 4–8 cell passages. RAW 264.7 cells were cultured in DMEM supplemented with 10% heat-inactivated fetal bovine serum, 100 U/mL penicillin, and 100 µg/mL streptomycin. The experiments were repeated 3 times, and the results are expressed as the mean  ±  SEM. Statistical significance (*p* < 0.05) was determined by one-way ANOVA followed by Tukey’s test for post hoc multivariate analysis. 

### 2.6. Western Blot Analysis 

We used the following antibodies: anti-iNOS (Abcam, Waltham, MA, USA, cat. ab15323) primary antibody followed by anti-rabbit IgG H&-HRP (Abcam, USA, cat. ab6721) secondary antibody and anti-GAPDH (Cell Signaling, USA, cat. 14C10) primary antibody followed by anti-rabbit IgG-HRP (Cell Signaling, USA, cat. 7074) secondary antibody. Macrophages were pelleted for 5 min at 600 g, resuspended in 1X loading buffer with Halt™ Protease Inhibitor Cocktail, and boiled for 5 min. The lysates were resolved on SDS-PAGE and blotted (Trans-Blot^®^ Turbo™) to an LF PVDF membrane. Blots were blocked in 5% fat-free milk in TBST at room temperature for 2 h. Subsequently, blots were incubated with primary antibodies at a 1:1000 dilution in blocking solution overnight at 4 °C. After washing 3 times (20 min each wash) in TBST, blots were incubated with a 1:5000 dilution of secondary antibodies and washed 3 times (20 min each wash) in TBST; protein bands were visualized with a ChemiDoc Imaging System (Bio-Rad, USA) using SuperSignal™ West Pico Chemiluminescent Substrate (Thermo Fisher, USA).

### 2.7. Immunostaining and Organelle Distribution Analysis

We used the following antibodies: anti-TOMM20 (Abcam, USA, cat. ab56783); anti-GM-130 (Abcam, USA, cat. ab52649); anti KDEL (Abcam, USA, cat. ab12223). Control and treated macrophages seeded on chamber slides (0.4 × 10^6^/slide) were fixed in 4% formaldehyde in PBS with 0.05% Triton X-100. After washing in PBS–Tween 20, fixed cells were blocked in casein blocking buffer (Bio-Rad, USA) with 0.05% Tween 20 and subsequently incubated in blocking buffer with a 1:200 dilution of primary antibodies and rhodamine–phalloidin overnight at 4 °C. Subsequently, after extensive washing in PBS–Tween 20, cells were mounted in ProLong^®^ Diamond Antifade with DAPI and observed under a Nikon fluorescence microscope. All experiments were performed in triplicate. The difference in organelle distribution between untreated and treated cells was calculated using the fluorescent area (normalized to total cell area using actin fluorescence) and corrected total cell fluorescence of individual cells using the image-processing ImageJ program. 

### 2.8. Flow Cytometry

The following antibodies were used for the flow cytometry analysis: purified anti-mouse CD16/32 antibody (FC Blocker) (Invitrogen, USA, cat. 14-0161-86), rat IgG (Invitrogen, USA, cat. 02-9602), Alexa Fluor 488 anti-mouse CD80, 16-10A1 (BioLegend, San Diego, CA, USA, #104716), Alexa Fluor 488 Armenian hamster IgG isotype antibody, HTK888 (BioLegend, USA, cat. 400923), and DAPI FluoroPure (Invitrogen, USA, cat. D21490). The flow cytometry was performed according to a previously used protocol [39].

### 2.9. Macrophage Polarization

For in vitro polarization, RAW 264.7 cells were cultured in DMEM supplemented with 10% heat-inactivated FBS, 100 U/mL penicillin, and 100 µg/mL streptomycin until passage 4 before subjecting them to polarization treatment. To polarize macrophages into the M1 phenotype, cells were incubated for 24 h with 100 ng/mL of LPS (Sigma–Aldrich, St. Louis, MO, USA, cat. L2654) and 20 ng/mL of IFN-γ (BioLegend, USA, cat. 575302). To polarize into the M2 phenotype, cells were incubated for 24 h with 20 ng/mL of IL-4 (BioLegend, USA, cat. 574302) and 20 ng/mL of IL-13 (BioLegend, USA, cat. 575904). Subsequently, the cell media were changed to normal DMEM media (with FBS, penicillin, and streptomycin) and cells were incubated for another 24 h. After 24 h of incubation, the cells were harvested and analyzed for the expression of M1- or M2-associated markers.

## 3. Results

### 3.1. The Effect of the Magnetic Field on Macrophages

**Figure 5:**
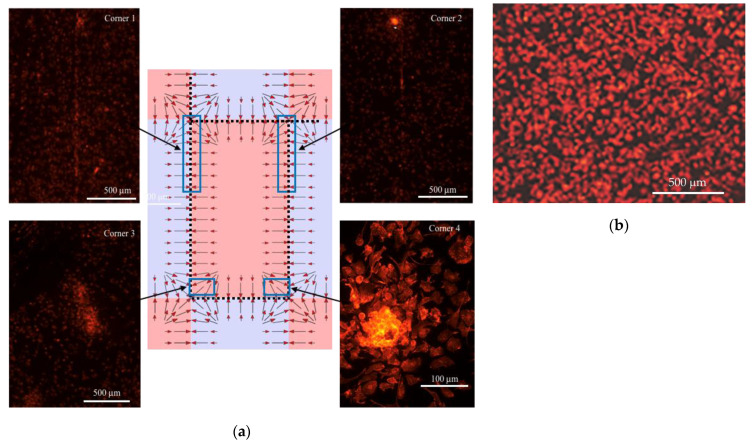
Pattern of the macrophages’ density in the magnetic field with no contrast agent added. (**a**) Macrophages were grouped above the central magnet corners and along the edges according to the magnetic gradient field and generated mechanical force patterns. The magnitude of the field gradients was the largest along the central magnet edges. The arrows show the direction of the mechanical force acting on the macrophages. (**b**) evenly distributed macrophages grown without magnetic field applied (stained red with Rhodamine phalloidin for actin) are shown as a control image.

**Figure 6:**
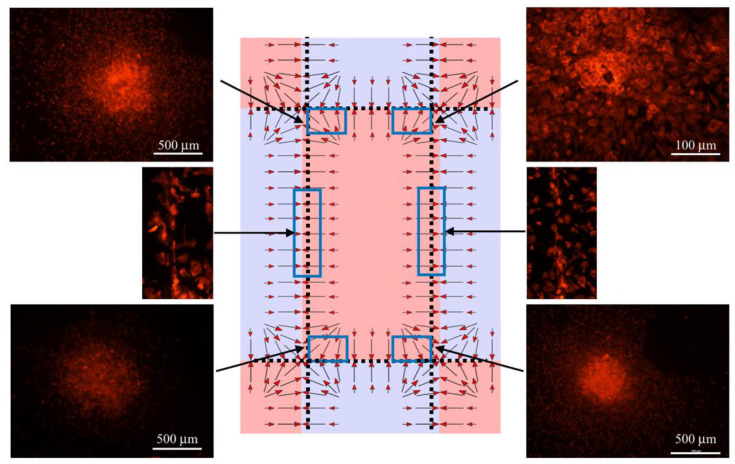
Pattern of the macrophages’ density in the magnetic field with contrast agent added. The macrophages were also grouped in the central magnet corners and along the edges according to the magnetic gradient field generated by the mechanical force pattern. Due to the paramagnetic properties of the contrast agent, the forces acting on the macrophages were significantly enhanced, which resulted in a much higher concentration of macrophages grouped in the corners and along the magnet edges compared to the no-contrast-agent case.

The analysis of the macrophage density distribution above the magnets (depicted in Figure 2) showed that without the magnetic field applied, the cells were evenly distributed; however, after application of the magnetic field, the macrophage concentration was found to be highly nonuniform after 48 h. As can be seen in Figure 5, they moved to and aggregated at the corners of the central magnet. In addition, the changes in the macrophages’ positions and their aggregation were highly enhanced by the presence of the gadolinium-based MRI contrast agent Dotarem as shown in Figure 6. The number of macrophages aggregated in the corners was 18 times higher (1800) than when the contrast agent was not added.

We calculated the translational force pattern, as shown in Figure 5 and Figure 6, and we simulated the movement of the cells for two directions of the F_mag_ (Equation (1)) force as determined by either the positive or negative Δχ susceptibility differences. Simulations showed that depending on the sign, the cells either will gather close to the N–S interface line or in the magnet center area. In these simulations, the cells were represented as spherical particles. The mass of each particle was selected to be equal to that of a cell, i.e., approximately 7–8 × 10^−12^ g. We compared the results of this simulation with the behavior of mouse macrophages exposed to magnetic field gradients [35]. We found a very good correlation between the calculated directions of the magnetic field-induced forces and experimentally observed alignment of the macrophages exposed to the gradients created by multiple permanent magnets as shown in Figure 5 and Figure 6.

The finding that the magnetic force determines the distribution of the cells in vitro suggests that, also in the in vivo situation, very strong magnetic field/magnetic field gradients generated by MRI may influence cell distribution and movement within the body. Additionally, the presence of gadolinium-based contrast agents exacerbated the magnetic field effect. These effects would potentially have a great impact on all “free-moving” cells such as immune cells in the animal or human exposed to MRI and gadolinium-based contrast agents.

We were also interested in the effects of Dotarem alone on the macrophage morphology and cellular and molecular components.

### 3.2. Dotarem Caused Cell Elongation and Redistribution of Cell Organelles

**Figure 7:**
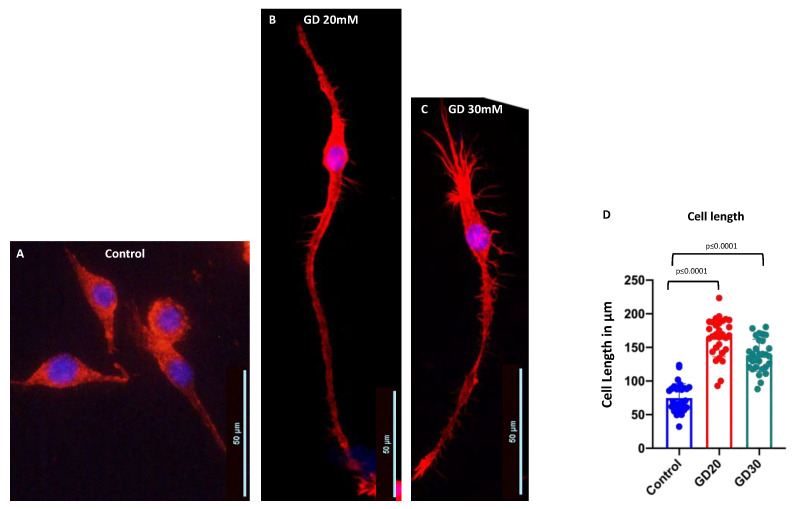
Gadolinium-based MRI contrast Dotarem caused cell elongation. (**A**–**C**) The images of RAW 264.7 macrophages stained with rhodamine–phalloidin (red) for actin. The nuclei were stained with DAPI (blue). (**A**) Control, untreated cells were slightly elongated. (**B**,**C**) The cells treated with 20 or 30 mM Dotarem (GD20 and GD30, **B**,**C**) were highly elongated. (**D**) Graph shows that the differences in cell length between the control and the Dotarem (GD)-treated cells calculated from three experiments were statistically highly significant.

The presence of Dotarem in the cell culture medium caused statistically significant elongation of macrophages (Figure 7). While the control macrophages grown in the absence of Dotarem were slightly elongated (approximately 50–70 µm in length) with a low number of highly elongated cells (approximately 100 µm in length) (Figure 7A,D), and the cells grown in 20 or 30 mM Dotarem-supplemented medium were highly elongated (over 150 µm in length) (Figure 7B–D). Although the exposure of macrophages to the magnetic field gradient or 20 mM Dotarem did not affect cell viability, the presence of 30 mM Dotarem was slightly toxic, decreasing cell number by approximately 30%.

**Figure 8:**
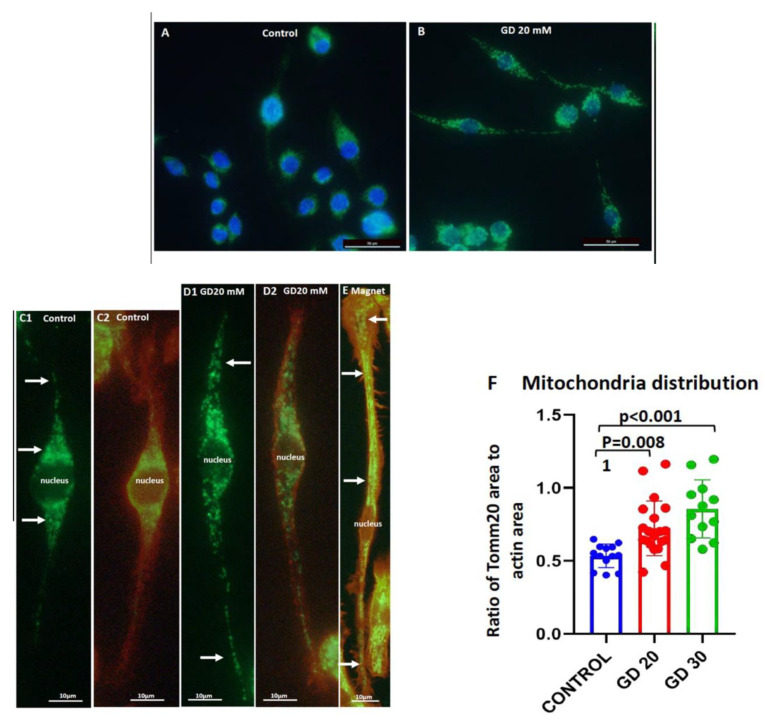
Dotarem and the magnetic field gradient caused redistribution of mitochondria. (**A**–**D**) Images of RAW 264.7 macrophages untreated (control) and treated with 20 and 30 mM Dotarem (GD20, GD30) stained with rhodamine–phalloidin (red) for actin and immunostained with mitochondrial marker Tomm20 (green) antibody. The nuclei were stained with DAPI (blue). Panels (**A**,**B**,**C2**) are merged images. (**E**) A mouse peritoneal macrophage that was grown on the magnet setup described by Wosik et al. (2018) and immunostained with mitochondrial marker COX4 (green) antibody in the merged image. In the control, the untreated cells of the mitochondria were located around and in the close vicinity of the nucleus (arrows) with very infrequent mitochondria observed in cell extensions. In the Dotarem-treated or magnetic field gradient exposed macrophages, mitochondria were distributed toward the cell extensions (arrows). (**F**) The graph shows that the differences in the mitochondria’s distribution (calculated from three experiments) between the control and the Dotarem (GD)-treated cells were statistically highly significant.

Knowing that the cell length and shape reflect actin distribution/dynamics and polymerization status, these findings indicate that Dotarem affects (directly or indirectly) the actin cytoskeleton of macrophages. Because the actin cytoskeleton also plays a role in anchoring cell organelles in their proper position within the cell, which is crucial for cell identity and functioning [40,41], we also studied the effect of Dotarem on the distribution of mitochondria, Golgi complex, and endoplasmic reticulum (ER) in RAW 264.4 macrophages. To study mitochondria distribution, we immunostained macrophages with the mitochondrial marker TOMM20 antibody against mitochondrial import receptor subunit TOM20 [42]. We found that while in the control macrophages, the mitochondria were mainly aggregated around the cell nucleus with very little mitochondria in cell extensions (Figure 8A,C1,C2,F), in the Dotarem-treated macrophages, mitochondria formed the clumpy aggregates rarely observed in the untreated cells and were redistributed away from the nucleus and toward the cell extensions (Figure 8B,D1,D2,F).

Interestingly, a similar redistribution of mitochondria was also observed in mouse peritoneal macrophages exposed to the magnetic field gradient (Figure 8E). To be sure that the redistribution of the mitochondria was not just a simple consequence of cell elongation, we paid special attention to those control macrophages that were highly elongated. We found that even in the elongated control macrophages, mitochondria were concentrated around the nucleus and in the cell body with a majority of them away from the cell tail (Figure 8C1,2) and that the changes in mitochondria’s distribution were statistically highly significant (Figure 8F).

**Figure 9:**
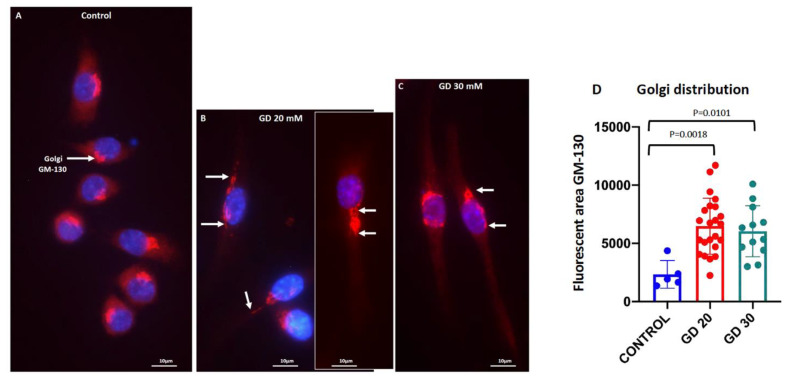
Dotarem caused a redistribution of the Golgi complex. (**A**–**C**) Images of RAW 264.7 macrophages untreated (control) and treated with 20 and 30 mM Dotarem (GD20, GD30) immunostained with the Golgi marker GM-130 (red) antibody. Nuclei were stained blue with DAPI. All are merged images. (**A**) In the untreated control cells, the Golgi formed a compact structure located to one side of the nucleus (arrow). (**B**,**C**) In the Dotarem-treated cells, the Golgi was distributed within the larger area in the vicinity of the nucleus and/or around the nucleus (arrows). Panel (**B**) is combined from two separate images. (**D**) The graph shows that the differences (calculated from three experiments) in the Golgi distribution between the control and the Dotarem (GD)-treated cells were statistically highly significant.

We also looked at the effect of Dotarem on the distribution of the Golgi complex. The immunostaining with the Golgi marker GM-130 antibody against a cis-Golgi matrix protein GM130 [43] showed that in the untreated macrophages, the Golgi complex formed a tight aggregate to one side of the nucleus (Figure 9A,D). In contrast, in the Dotarem-treated cells, the Golgi complex occupied a much broader area and was distributed around the nucleus or redistributed away from the nucleus (Figure 9B–D). These changes in the Golgi distribution between the control and the Dotarem-treated cells were statistically highly significant (Figure 9D). In contrast, our previous studies on the effect of the magnetic field gradient on the Golgi complex in peritoneal mouse macrophages showed that these macrophages lost a well-defined Golgi complex and had only a very faint and dispersed staining in the cytoplasm [27].

**Figure 10:**
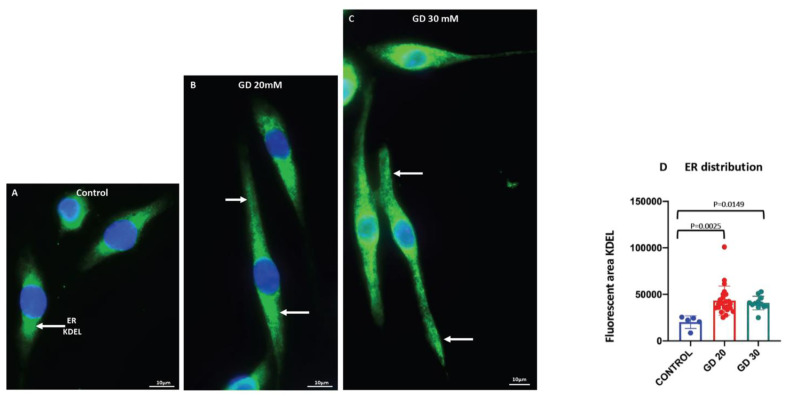
Dotarem caused a redistribution of the ER. (**A**–**C**) Images of RAW 264.7 macrophages untreated (control) and treated with 20 and 30 mM Dotarem (GD20, GD30) immunostained with the endoplasmic reticulum (ER) marker KDEL (green) antibody. Nuclei are stained blue with DAPI. All are merged images. (**A**) In the untreated control cells, the ER was located in the cell body in the vicinity of the nucleus (arrow). (**B**,**C**) In the Dotarem-treated cells, the ER was distributed within the larger area and in the extensions (arrows). (**D**) The graph shows that the differences (calculated from three experiments) in ER distribution between the control and the Dotarem (GD)-treated cells were statistically highly significant.

We also found that there was a statistically significant change in the distribution of the endoplasmic reticulum (ER) in the Dotarem-treated cells. The immunostaining with the ER marker of the KDEL antibody against the endoplasmic reticulum protein retention receptor showed that while in the control cells, the ER was concentrated in the cell body and the vicinity of the nucleus (Figure 10A,D); the treatment with Dotarem caused redistribution of the ER toward the cell extensions (Figure 10B–D). 

**Figure 11:**
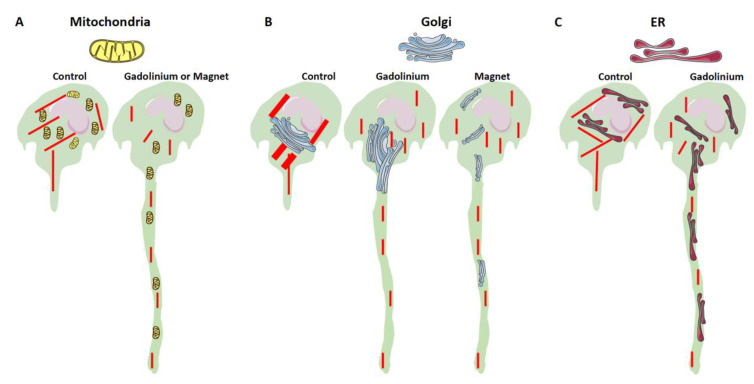
Diagram summarizing the effects of Dotarem and the magnetic field gradient on the distribution of organelles. (**A**–**C**) Distribution of mitochondria, Golgi, and ER in the control and treated cells. All these organelles were anchored at their proper subcellular locations by the actin filaments (depicted in red). Dotarem (gadolinium) treatment and magnetic field gradient exposure disrupted the actin cytoskeleton and changed the distribution of organelles. (**A**) Dotarem treatment and magnetic field gradient exposure had very similar effects on the distribution of mitochondria. (**B**) Dotarem treatment or magnetic field gradient exposure changed the Golgi distribution, but the effects were different. While Dotarem caused the extension of the Golgi marker-positive area, exposure to the magnetic field gradient caused, as described by Wosik et al. (2018), the dispersion of the Golgi complex. (**C**) Dotarem treatment caused a redistribution of the ER toward the cell extensions. There are no data on the effect of the magnetic field on ER distribution in the studied cells.

**Figure 12:**
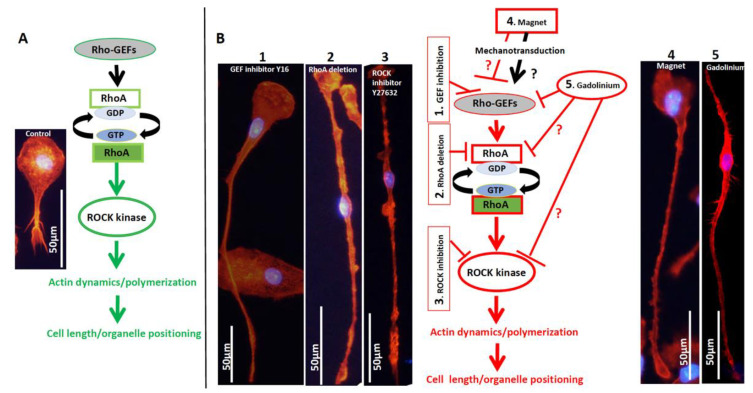
Hypothetical effects of RhoA pathway interference (using inhibitors, RhoA KO, gadolinium, and the magnetic field gradient) on the actin cytoskeleton and cell length. (**A**) The length of a typical MO (unstimulated) macrophage was usually below 100 μM. The cell morphology and distribution of the organelles were actin-dependent and regulated by the RhoA pathway. RhoA is activated by the RhoA-specific guanine nucleotide exchange factors (GEFs). Activated RhoA, through its downstream effector ROCK kinase, regulates actin dynamic and polymerization which, in turn, regulate cellular structure, distribution, and functions. (**B1**–**B3**) As we have shown before, the inhibition of RhoA-GEFs by Y16 or Rhosin [46], macrophage-specific RhoA knockout [47], or inhibition of ROCK by Y27632 [36] disrupted actin dynamics and polymerization, causing extreme macrophage elongation to >150 μM. (**B4**) The mechanotransduction-related effects of the magnetic field gradient on the cell membrane, Ca^2+^ channels, and GEFs, which are known to be activated by Ca^2+^, disrupted the normal functioning of the RhoA pathway, modulating actin dynamics, and causing cell elongation. (**B5**) There were indications that gadolinium may affect the function of GEFs through the blockade of the calcium channels, affecting Ca^2+^ influx and homeostasis [48,49] thus influencing the RhoA/actin pathway and causing cell elongation. The possible effects of gadolinium on other components of the RhoA pathway are unknown.

A diagram summarizing the effect of Dotarem on cell length and organelle distribution is shown in Figure 11. All these findings indicate that the gadolinium-based contrast agent Dotarem affects the geometrical morphotype of the cell and the anchoring of its organelles. These processes depend on the proper polymerization and distribution of actin filaments and the globular (G) versus filamentous (F) actin dynamics. Interestingly, as we showed before [35], nearly identical phenotypical changes, such as elongation and redistribution of the organelles away from the nucleus, occurred in macrophages exposed to the magnetic field gradient. This study also showed that exposure to the magnetic field gradient caused clustering of the cation channel receptors TRPM2, which are the Ca^2+^-permeable cation channels from the ion transport protein family in the vicinity of the nucleus [27]. We hypothesized that the clustering may render the channels nonfunctional, which may disrupt calcium homeostasis and calcium-dependent processes such as actin polymerization [27,35]. It is well established that the actin dynamics and polymerization status are regulated by the small GTPase RhoA pathway [41]; thus, our findings on cell elongation and organelle redistribution suggest that the gadolinium (Dotarem) and magnetic field gradient may affect various components of the RhoA pathway (Figure 12, see Section 4).

Because it is known that the geometrical morphotype changes in the dynamics between the G and F actin and that the influx of G actin into the cell nucleus also has profound effects on the gene expression pattern [44,45], we also checked whether and how Dotarem affects the expression of macrophage signature genes. 

The macrophages have many functionally different subtypes including the unstimulated MO, the classically activated (inflammatory) M1, and the anti-inflammatory M2 macrophages [50]. The M1 and M2 subtypes, which can be easily created in vitro from the unstimulated MO macrophages by treatment with cytokines and stimulating factors (listed in Section 2), express different signature molecules [51,52].

**Figure 13:**
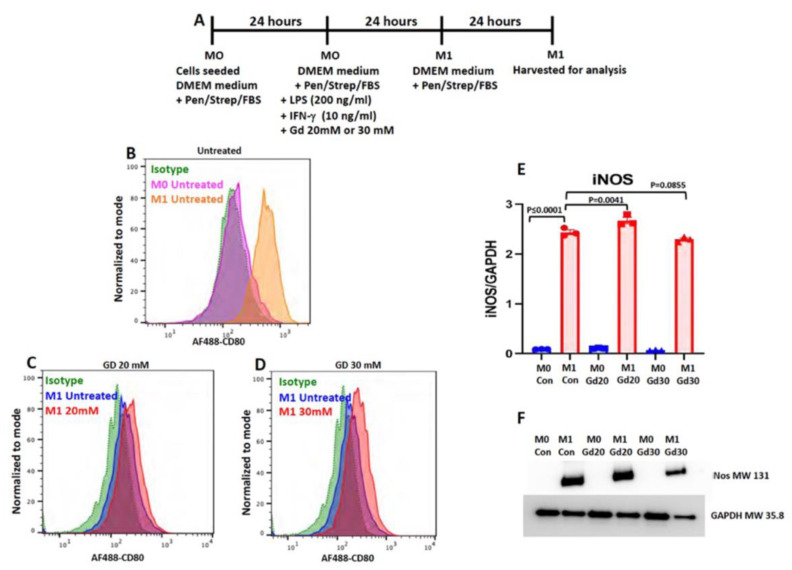
Dotarem upregulated the expression of pro-inflammatory (M1) markers. (**A**) The scheme and timeline of M1 polarization and Dotarem (gadolinium, Gd) treatment. (**B**–**D**) Flow cytometry analysis of the expression of M1 marker CD80 showed the upregulation of the expression in gadolinium-treated cells. To make the results more accurate by taking into account the differences in the number of living cells between the experimental groups, we normalized the cell count to the percentage mode. (**E**,**F**) Western blot analysis of M1 marker iNOS expression showed statistically significant upregulation of the expression in gadolinium-treated cells.

**Figure 14:**
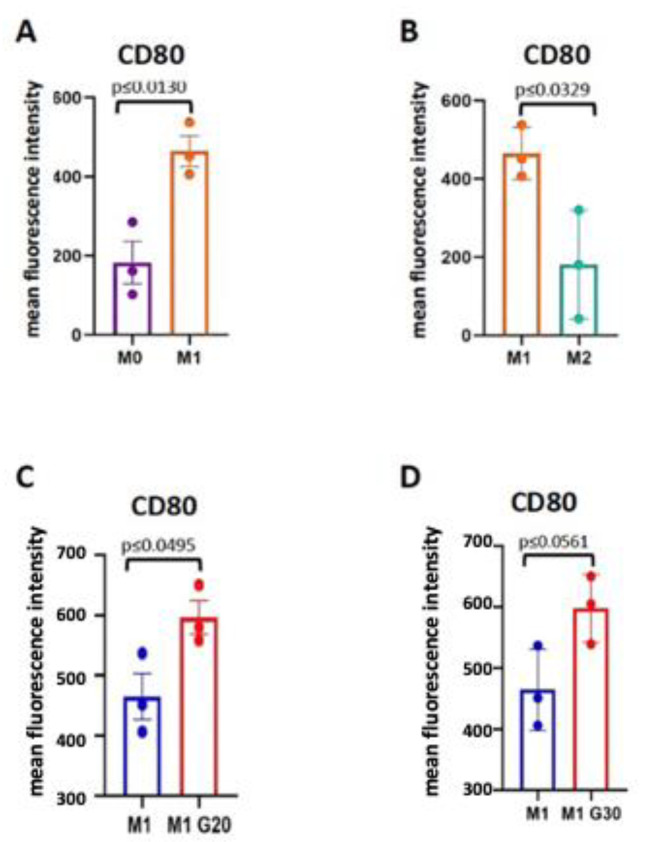
Specificity of expression of M1 marker CD80. (**A**–**D**) Comparison of CD80 expression levels between MO, M1, and M2 macrophages treated and untreated with gadolinium showed that the high expression of CD80 was specific for the M1 macrophages.

For example, one of the markers of M1 macrophages is the inducible nitric oxide synthase (iNos) [20,39] that catalyzes the production of nitric oxide (NO) from L-arginine. Another is the co-stimulatory molecule CD80 (cluster of differentiation 80), which is the type I membrane protein from the immunoglobulin superfamily [52]. Western blot of iNos and flow cytometry analysis of CD80 protein expression levels showed that the expression of both of these markers was upregulated in the Dotarem-treated cells (Figure 13 and Figure 14). 

**Figure 15:**
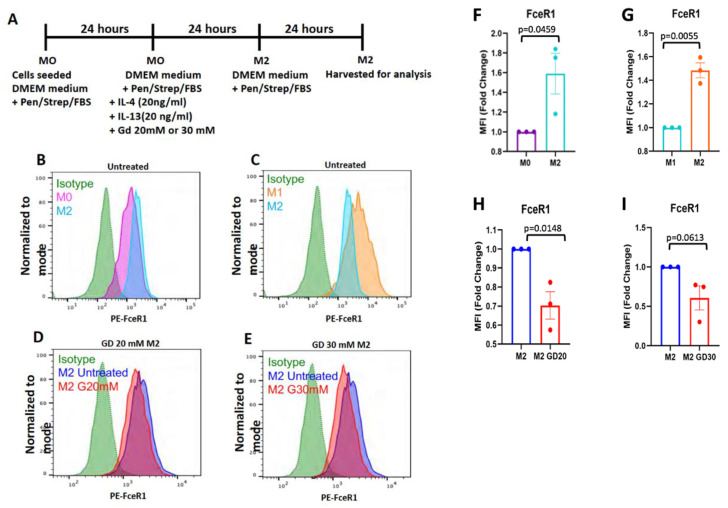
Dotarem downregulated the expression of the anti-inflammatory M2 marker FceR1. (**A**) The scheme and timeline of M2 polarization and Dotarem (gadolinium, Gd) treatment. (**B**–**E**) Flow cytometry analysis indicated that the high expression of FceR1 was characteristic for M2 macrophages and that gadolinium treatment decreased FceR1 expression. (**F**–**I**) Graphs from the flow cytometry data showed that the upregulation of FceR1 expression in M2 macrophages and its downregulation upon Dotarem treatment were statistically significant.

**Figure 16:**
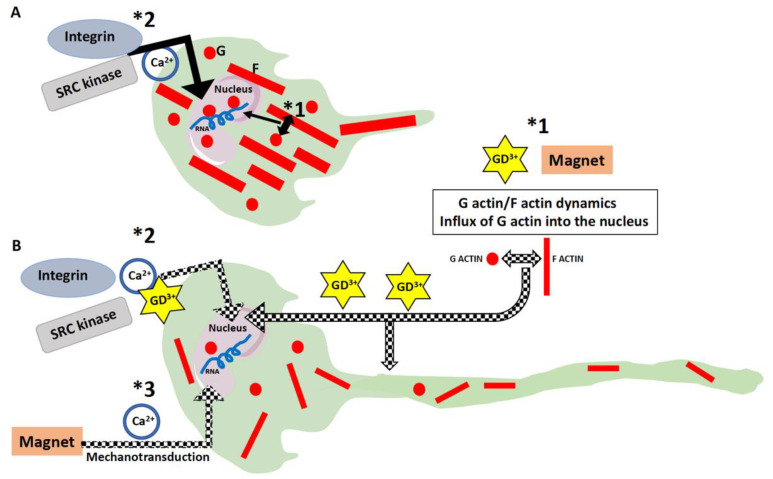
Diagram of the hypothetical effects of gadolinium and the magnetic field gradient on gene expression. (**A**) In the control cell, the balance between the globular (G) and filamentous (F) actin and the influx of G actin into the nucleus (*1) were precisely controlled. Nuclear G actin participated in the regulation of transcription by binding to and activating RNA polymerases and by modifying chromatin compaction. The nuclear processes, including transcription, can also be modified by the calcium-dependent integrin and SRC kinase pathways (*2). (**B**) Exposure to gadolinium or the magnetic field gradient could disrupt G/F actin homeostasis (*1), affecting transcription and causing cell elongation. Gadolinium may also affect nuclear functions through the disruption of calcium signaling and the integrin and SRC pathways (*2). Magnetic field gradient exposure may, through mechanotransduction/calcium-dependent signaling (*3), affect nuclear processes including gene transcription.

**Figure 17:**
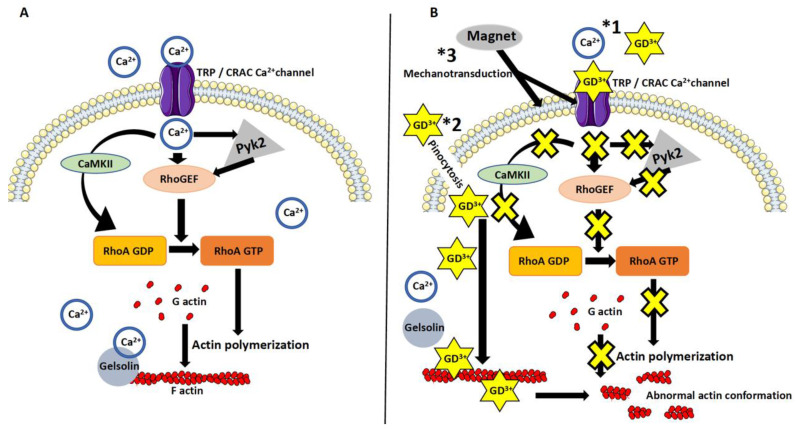
Diagram of the hypothetical effects of gadolinium and the magnetic field gradient on the RhoA pathway and actin dynamics. (**A**) In the untreated cells, calcium influx into the cell and calcium homeostasis were regulated through the TRP/CRAC ion channels. Calcium may regulate GEFs-dependent activation of RhoA through the proline-rich tyrosine kinase 2 (Pyk2) pathway or activate RhoA through the Ca(^2+^)/calmodulin-dependent protein kinase II (CaMKII) pathway. In turn, activated RhoA regulates G/F actin homeostasis and actin polymerization and, thus, all actin-dependent cell functions. Actin polymerization may also be affected directly through a calcium-regulated actin filament-capping protein gelsolin. (**B**) Extracellular gadolinium (*1) may block ion-exchange channels and consequently inhibit GEFs and all the downstream RhoA pathway-dependent processes. Gadolinium may also enter the cell through pinocytosis (*2) and directly affect actin polymerization and homeostasis by preventing binding of calcium to actin and/or gelsolin or directly altering actin filament conformation. The force created by the magnetic field gradient (*3) may, through mechanotransduction, affect ion-exchange channels that, in turn, will disrupt GEFs and RhoA activation and affect all actin-dependent cell functions.

This indicates that Dotarem treatment enhanced the pro-inflammatory M1 phenotype. We also checked by flow cytometry the expression of the anti-inflammatory M2 macrophage marker—the high-affinity IgE receptor FceR1. FceR1 controls the production of inflammatory mediators such as histamines [53,54]. Flow cytometry analysis showed that Dotarem treatment decreased the expression of FceR1 in M2 macrophages, thus downregulating the pro-inflammatory phenotype (Figure 15). In contrast to these findings, as we showed before, the macrophages exposed to the magnetic field gradient did not express the pro-inflammatory M1 marker iNos but, instead, increased the expression of the anti-inflammatory M2 marker Arg-1 [27,35]. Thus, the exposure of the macrophages to the gadolinium-based contrast agent Dotarem and the magnetic field gradient had the opposite effect on the expression of pro- and anti-inflammatory markers [20]. While gadolinium increased the pro-inflammatory and decreased the anti-inflammatory phenotypes, the magnetic field gradient inhibited the pro-inflammatory and increased the anti-inflammatory macrophage phenotypes. Our hypothesis as to how the gadolinium and magnetic field gradient may affect gene expression in macrophages is summarized in Figure 16 and Figure 17.

## 4. Discussion

We showed that the exposure of mouse macrophages to the magnetic field gradient in the presence of gadolinium-based MRI contrast agent Dotarem caused the aggregation of macrophages; treatment of macrophages with Dotarem affected their morphology, the distribution of organelles, and gene expression pattern, shifting the macrophage phenotype toward the pro-inflammatory. 

Our previous in vivo and in vitro studies on the role of macrophages in the development of chronic (long-term) rejection of transplanted organs showed that macrophage length, distribution of organelles, and gene expression are regulated by the RhoA pathway [36,41,46,47,55]. RhoA is a small GTPase that controls actin dynamics and polymerization [56]. The RhoA is activated by guanine nucleotide exchange factors (GEFs) that promote the exchange of RhoA-bound guanosine diphosphate (GDP) into guanosine triphosphate (GTP) [57]. Among many signaling molecules and pathways that can activate GEFs, one is calcium signaling [58]. The RhoA-specific GEFs can be regulated directly by the Ca^2+^ or through the Ca^2+^ mediated-activity of proline-rich tyrosine kinase 2 (PYK2) [59]. Another calcium-dependent pathway regulating GDP/GTP exchange during RhoA activation is the Ca^2+^/calmodulin-dependent protein kinase II (CaMKII) [60]. Once activated, the RhoA-GTP regulates the actin cytoskeleton through the activation of the downstream effector ROCK kinase (Figure 17A) [61]. 

We showed previously that the inhibition of RhoA-GEFs with Rhosin or Y16 [46], macrophage-specific RhoA deletion [55], or inhibition of ROCK [5] by Y27632 [36] caused extreme elongation of macrophages (they acquired the so-called hummingbird phenotype), redistribution of macrophage organelles and receptors, and affected gene transcription. We also showed that similar changes were induced in the macrophages exposed to the magnetic field gradient [35,41]. We also showed that magnetic field exposure caused aggregation of the TRPM2 (the cation channel transient receptor potential melastatin 2/Transient Receptor Potential Cation Channel Subfamily M Member 2) that is a Ca^2+^ channel regulating calcium homeostasis [62]. It is known that nonuniform magnetic fields can create a magnetic force that changes the ion-channel activity (such as Ca^2+^) at the cell membrane, conveying the mechanical stresses from the cell membrane to the cell interior in the process called mechanotransduction [34,63,64,65]. We hypothesized that TRPM2 clustering rendered them nonfunctional and disrupted Ca^2+^ homeostasis which, in turn, affected the RhoA pathway, which is Ca^2+^-dependent [60], and changed the macrophage actin cytoskeleton and actin-dependent functions [27,35]. In the present study, we showed that the gadolinium-based MRI contrast agent Dotarem had very similar effects on the macrophages to that described above (Figure 17B). We also showed that both gadolinium and the magnetic force gradient changed the expression of macrophage M1 and M2 markers. However, while gadolinium increased the pro-inflammatory (M1) and decreased the anti-inflammatory (M2) phenotypes, the magnetic field gradient inhibited the pro-inflammatory and increased the anti-inflammatory macrophage phenotypes. Recent studies indicate that the functions of the cell nucleus depend on the nuclear actin, which regulates chromatin remodeling and gene transcriptions [41] which, in turn, are regulated by calcium homeostasis. The dynamics between the G and F actin regulating the influx of G actin to the nucleus have a direct effect on nuclear functions and binding to and activating RNA polymerases regulates transcription (Figure 16) [41]. The nuclear processes, including transcription, are also regulated by the calcium-dependent integrin and SRC kinase pathways [66,67] (Figure 10). Based on all our data and information from the literature on the known effects of gadolinium Gd^3+^ on cellular processes, we formulated a hypothetical model as to how gadolinium may affect the RhoA pathway, causing the downstream changes in the actin cytoskeleton dynamics and its nuclear and cytoplasmic functions (Figure 16 and Figure 17). We postulate that extracellular gadolinium ions block calcium channels (pathway *1 in Figure 11B) [68,69], and the magnetic field gradient-created force, by affecting the cell membrane, changes the localization and/or permeability of calcium channels (pathway *3 in Figure 17B) [70], which affect calcium homeostasis and all downstream pathways and processes involved in RhoA/actin signaling (Figure 17B). Additionally, gadolinium can be taken by the cell through the pinocytosis (pathway*2 in Figure 17B) [71] and affects actin polymerization either by directly binding to the actin and changing its confirmation [49] or preventing the Ca^2+^-dependent gelsolin function by depleting calcium [72].

There are still many unanswered questions. What molecular pathways are involved in the similarities and differences in response to gadolinium and the magnetic field gradient? What would be (besides the cellular aggregation shown in this study) the molecular effects of the magnetic force gradient and the gadolinium when applied together (as in the MRI with the contrast agent present)? Although further in vivo studies are needed on the subject, our findings suggest that a clinical administration of Dotarem may not be as safe as previously thought, especially if, as recently found, gadolinium accumulates in tissue for a long time [73].

## Data Availability

On request.
